# A study on the natural history of scanning behaviour in patients with visual field defects after stroke

**DOI:** 10.1186/s12883-015-0321-5

**Published:** 2015-04-24

**Authors:** Tobias Loetscher, Celia Chen, Sophie Wignall, Andreas Bulling, Sabrina Hoppe, Owen Churches, Nicole A Thomas, Michael E R Nicholls, Andrew Lee

**Affiliations:** School of Psychology, University of South Australia, Adelaide, Australia; Department of Ophthalmology, Flinders University, Adelaide, Australia; School of Psychology, Flinders University, Adelaide, Australia; Max Planck Institute for Informatics, Saarbrucken, Germany; Flinders Comprehensive Stroke Centre, Flinders Medical Centre, Adelaide, Australia

**Keywords:** Hemianopia, Eye tracking, Longitudinal, Stroke, Walking, Dynamic assessment

## Abstract

**Background:**

A visual field defect (VFD) is a common consequence of stroke with a detrimental effect upon the survivors’ functional ability and quality of life. The identification of effective treatments for VFD is a key priority relating to life post-stroke. Understanding the natural evolution of scanning compensation over time may have important ramifications for the development of efficacious therapies.

The study aims to unravel the natural history of visual scanning behaviour in patients with VFD. The assessment of scanning patterns in the acute to chronic stages of stroke will reveal who does and does not learn to compensate for vision loss.

**Methods/Design:**

Eye-tracking glasses are used to delineate eye movements in a cohort of 100 stroke patients immediately after stroke, and additionally at 6 and 12 months post-stroke. The longitudinal study will assess eye movements in static (sitting) and dynamic (walking) conditions.

The primary outcome constitutes the change of lateral eye movements from the acute to chronic stages of stroke. Secondary outcomes include changes of lateral eye movements over time as a function of subgroup characteristics, such as side of VFD, stroke location, stroke severity and cognitive functioning.

**Discussion:**

The longitudinal comparison of patients who do and do not learn compensatory scanning techniques may reveal important prognostic markers of natural recovery. Importantly, it may also help to determine the most effective treatment window for visual rehabilitation.

## Background

Visual field defects (VFD) occur in 20 - 57% of strokes [1], negatively impacting the survivors’ functional ability and quality of life [1,2]. A stroke survivor may demonstrate difficulties with reading and navigation, and is at increased risk of falling. This, in turn, can induce anxiety, a marked loss of confidence, social isolation and depression [1,2].

The identification of the most efficacious treatments for visual problems has been ranked among the top six research priorities concerning life post-stroke [3]. Up to 50% of patients display no spontaneous improvement or are left with residual visual defects [4]. The question of how to rehabilitate vision in these patients remains to be answered [1,5].

While the learning of compensatory scanning techniques towards the side of the VFD is seen as a promising treatment [1,6,7], the natural evolution of VFD compensation over time has not been thoroughly examined. Existing studies of scanning patterns in VFD are flawed by their cross sectional nature (e.g., [8,9]) and/or by the presentation of visual stimuli on stationary screens or projectors. Such stationary stimuli fail to replicate real world situations wherein patients are recurrently faced with moving environments [10]. The lack of longitudinal data makes it difficult to determine the subset of patients who would most benefit from VFD rehabilitation, as well as the optimal duration for such therapy.

We aim to address the issue of the natural history of visual scanning behaviour in patients with VFD. Scanning patterns will be assessed in static (sitting) and dynamic (walking) conditions with eye-tracking glasses. Understanding the natural evolution of scanning compensation over time may have important ramifications for the development of effective treatments.

## Methods/Design

This is an observational, longitudinal study of a cohort of 100 stroke survivors. All patients will receive standard care. The study has been approved by the Southern Adelaide Clinical Human Research Ethics Committee.

### Patient population

Fifty stroke patients with visual field defects and a cohort of 50 stroke patients without visual field defects will be recruited at the Flinders Comprehensive Stroke Centre.

### Inclusion and exclusion criteria

Subjects will be older than 18 years of age and have the capacity to provide full and informed consent. There is no upper age limit. All patients will have a clinical diagnosis of stroke, defined as the presence of a focal neurological defect due to vascular cause that is present at 24 hours or beyond. Brain imaging (CT or MRI) will confirm the nature of the stroke.

Patients must be able to mobilise independently or with minimal assistance during the recruitment period, evidenced by a post-stroke modified Rankin Score greater than three. Moreover, patients with significant medical co-morbidities which preclude participation in the comprehensive clinical and cognitive testing will be excluded.

### Schedule of assessments

Each patient will be assessed at three different time periods:

- Acute: within the first two weeks of stroke.
- Subacute: three to six months post-stroke. This is the first follow-up period.
- Chronic: 12-15 months post-stroke. This is the final follow-up period.

### Assessments

Stroke, ophthalmic, cognitive and eye tracking assessments will be performed (see Table 1).

**Table 1:** **Schedule and list of assessments**

	**Acute phase (0-2 weeks)**	**Subacute (3-6 months)**	**Chronic (1-2 years)**
**Clinical stroke assessment**			
• History	X		
• Clinical examination	X		X
• Risk factor evaluation	X		
• Modified Rankin	X	X	X
• CT brain	X		
• MRI brain		X	X
**Ophthalmic evaluation**			
• Visual acuity	X	X	X
• Humphrey’s visual field	X	X	X
**Cognitive assessment**			
• Addenbroke’s cognitive evaluation	X	X	X
• Standardized reading assessment	X	X	X
• Spatial neglect screening	X	X	X
**Eye movements during search task**			
• Stationary search task	X	X	X
• Dynamic search task	X	X	X

#### Clinical stroke assessment

The stroke clinical assessment is part of the standard practice outlined in the Flinders Comprehensive Stroke Centre care protocol. Imaging of the brain is performed on admission and often at discharge, or within the first three months following the stroke. All patients, where possible, are followed up at six-and 12-months post discharge. During this time, systemic clinical workup including an assessment of improvements in the patients’ clinical condition, vascular risk factors, and emotional state is performed (see Table 1).

#### Ophthalmic assessment

The ophthalmic examination will include visual acuity and Humphrey’s automated visual field testing.

#### Cognitive assessment

Patients’ cognitive functions will be screened with the Addenbrooke’s Cognitive Examination (ACE-III, [11]). Patients will also be assessed on a standardized reading performance task (IResT, [12]) and a series of visuospatial tests (Rey-Osterreith Complex Figure [13], Line Bisection (9 lines of 16cm length, presented separately on an A4 horizontal Sheet), and Bells cancellation task [14]). Performance in the individual visuospatial tests will be standardised using established norms. These standardised scores will then be averaged to calculate a domain score of a patient’s visuospatial ability.

#### Eye tracking assessment

Patients will complete both a stationary and dynamic visual search task while their eye movements are tracked with SMI Eye Tracking Glasses (SensoMotoric Instruments GmbH, Berlin, Germany). Eye movements will be separately analysed for each task.

The stationary task is adapted from [15], and is designed to assess the common attentional problem of identifying a particular face or individual within a crowd. The stimuli are derived from the popular “Where’s Wally” children’s book series, and consist of cluttered, densely populated illustrations [16]. Hidden within each illustration is a distinctly dressed character, named “Wally”. The stimulus set consists of five such illustrations presented sequentially on a computer screen. Each stimulus will be displayed in its original and mirror-reversed orientation in order to control for visual properties that could influence lateral eye movements (e.g., side of target, possible lateral differences in salience and clutters). Patients will have 60 seconds to locate Wally within each illustration, after which the next stimulus will be presented. The arbitrary time-out of 60 seconds was chosen to restrain the maximum task duration. The patients’ eye movements, reaction times and the number of missed targets as a function of target side will be measured. Head movements will not be constrained for the duration of the task.

The dynamic search task aims to assess eye movements during walking. Patients will walk a standardized mobility assessment course within the ward corridors (see [17]), along which twenty yellow targets (measuring 100 mm × 100 mm) will be placed. The targets will be presented at different heights and against different backgrounds, and will be evenly distributed to the left and right sides of space. Patients will be instructed to indicate the position of the targets when walking the course both in clockwise and anti-clockwise directions (order counterbalanced across patients). Importantly, the examiner will walk closely behind the patient and, when necessary, will intervene to prevent collisions. Eye movements and the number of omitted targets as a function of target side will be measured.

### Primary outcomes

The primary outcome constitutes the change of lateral eye movements from the acute to chronic stages of stroke. The total time (seconds), number of fixations, number of saccades and saccade amplitudes in the contralesional field relative to the ipsilesional side of space will be used to measure lateral eye movements. We expect to find a relative increase of time spent, fixations and number of saccades in the contralesional field over time.

### Secondary outcomes

Secondary outcomes include changes of lateral eye movements over time as a function of subgroup characteristics. We will determine whether side of VFD, stroke location, stroke severity, macular sparing, presence of spatial neglect and cognitive functioning influence changes of eye movements from the acute to chronic stages of stroke.

### Statistical analyses

Begaze analysis software (SensoMotoric Instruments GmbH, Berlin, Germany) will be used to calculate the following quotients of lateral eye movements for each patient: (1) total time spent looking towards the blind side divided by the time looking towards the intact side of space and (2) number of fixations towards blind side divided by number of fixations in intact side. Statistical analyses will be performed using SPSS software (SPSS Inc, Chicago, Illinois, USA).

For continuous variables (expressed as a mean and standard deviation) the patients with VFD and patients without VFD will be compared using a mixed-design analysis of variance. In the case of imbalanced group characteristics (e.g., age) we will include them as co-variants in the analysis. An alpha of 0.05 will be considered significant.

### Sample size estimates

The study is powered with respect to the primary outcome. Based on previous research [18], we expect a large effect when comparing the relative time (i.e. cumulative fixation duration) of left and right-sided space explorations in patients with VFD and control patients. A power analysis (Cohen’s d > 1.00 [18], alpha = .05, and power = 0.8) suggests that a sample size of 34 participants is sufficient. Our sample of 100 participants is thus relatively large, allowing for attrition in the follow-up studies and subgroup analyses.

## Conclusions

Stroke survivors, caregivers and health professionals consider the identification of effective treatments for visual loss as one of the six top priorities relating to life post- stroke [3]. The results of this study will reveal the natural history of scanning behaviour in patients with visual field loss. The repeated assessments will permit an examination of the degree of natural recovery (restoration). A longitudinal comparison of patients who do and do not learn compensatory scanning techniques may reveal prognostic markers of recovery (functional compensation). Moreover, understanding the trajectory of natural eye movement patterns may help to determine the most effective treatment window for visual rehabilitation [4].
